# Impairments of Syntactic Comprehension in Korean and the Location of Ischemic Stroke Lesions: A Voxel-Based Lesion-Symptom Mapping Study

**DOI:** 10.3233/BEN-2009-0254

**Published:** 2010-06-10

**Authors:** M. J. Kim, H. A. Jeon, K. M. Lee

**Affiliations:** ^1^Department of NeurologySamsung Medical CenterSungkyunkwan University School of MedicineSeoulRepublic of Korea; ^2^Neuroscience Research InstituteGachon Medical SchoolIncheonRepublic of Korea; ^3^Department of NeurologySeoul National University HospitalSeoulRepublic of Korea

**Keywords:** Language tests, comprehension, aphasia, brain mapping, stroke, linguistics

## Abstract

In order to assess the capacity of neurological patients to process syntactic features unique to Korean, such as the heavy dependence of parsing on syntactic morphemes rather than the word order in a sentence, the Korean Syntactic Comprehension Test (KSCT) was newly developed. To examine the correlation between lesion locations and the test performance, we did voxel-based lesion-symptom mapping (VLSM) analysis in a group of 39 patients with ischemic stroke. As a result, KSCT scores of the aphasic patients were significantly lower than those of 18 normal subjects. Within the patient group, VLSM analysis showed significant association between lower KSCT performance and the lesions mainly located in left perisylvian area and anterior temporal lobe. The KSCT results were also closely correlated with the results of two subtests in the Korean version-the Western Aphasia Battery. We conclude therefore that brain localization of syntactic comprehension in Korean native speakers is similar to that in other language speakers, despite the unique features of the Korean syntax, and that the KSCT will be of diagnostic value in assessing left fronto-temporal functions in Korean patients.

## 1. Introduction

Broca's aphasia is often regarded as a primarily expressive disorder, since the patients' comprehension appears to be relatively intact in comparison with their disorganized speech [1]. However, a number of important studies demonstrated that when all semantic and pragmatic cues to meaning are eliminated so that comprehension must rely on syntactic structure alone, Broca's aphasics perform very poorly [2,3]. The reason is that syntactic comprehension also depends on a temporo-frontal network, including the left inferior frontal and left anterior/superior temporal region. The former region includes left inferior frontal gyrus, frontal operculum, insular cortex and adjacent basal ganglia, and the latter region includes anterior, middle, and posterior portion of left superior temporal gyrus although the middle portion appears to be more involved in semantic processing [4].

While this localization for syntactic comprehension might hold true for the Korean language as well, there are some reasons to suspect this may not be the case: Korean syntactic structure has features that are different from European languages which were most often used

ISSN 0953-4180/09,10/$17.50 © 2009/2010 – IOS Press and the authors. All rights reserved

Table 1
Examples of representative characteristics of Korean syntactic structure

| | Examples | |
|---|---|---|
| Action of Postpositional words | (1) So-**ka** kae-**lul** jjoc-nun-ta.<br>cow-Nom dog-Acc chase-SE<br>(2) 'The cow chases the dog.' | (3) Kae-**lul** so-**ka** jjoc-nun-ta.<br>dog-Acc cow-Nom chase-SE<br>(4) 'The dog chases the cow.' |
| Action of Postpositional words and Sentential endings | (5) So-**ka** kae-**lul** jjoc-**nun**-ta.<br>cow-Nom dog-Acc chase-SE<br>(6) 'The cow chases the dog.' | (7)So-**ekey** kae-**ka** jjoc-**ki**-**n**-ta.<br>cow-Dat dog-Nom be.chase-SE<br>(8) 'The dog is chased by the cow.' |

Nom: nominative. Acc: accusative. SE: sentence ender. Dat: dative. Postpositional words: **-ka, -lul, -ekey.** Sentential endings: **-nun, -ki-n-**.

in previous studies on the issue. The thematic roles and sentential forms in Korean sentences are heavily dependent on syntactic morphemes such as postpositional words and sentential endings. Sentential meanings are preserved even if the word order is changed, as long as the morphemes are placed properly. This is in contrast to many other languages in which the thematic roles are assigned almost exclusively by word order: The meaning is changed completely or the sentence becomes agrammatical when the word order is changed in such languages. Owing to this distinct property, Korean sentences have been used in a few studies about linguistic models of grammatic comprehension deficit [5, 6]. In those studies, they used Korean to investigate the effect of scrambled words within a sentence since such manipulation was relatively limited in other languages.

Examples from Korean and English are given in Table 1. The meaning of Korean sentence (3) is similar to (1) despite the order of the words denoting dog and cow were reversed each other. Postpositional words like "-ka" and "-lul" within those sentences make the nouns as a subject and an object, respectively, regardless of their order within the sentence. In contrast, the meaning of English sentence (4) is the opposite of (2) – the roles of the words are changed when their order is reversed. Another example demonstrating the importance of sentential endings in Korean is given in the comparison between the sentence (5) and (7). The Korean sentence (5) can be changed from an active form to a passive form in sentence (7) while the order of the words is maintained constantly. This is from the changes of postpositional words (from "-ka" and "-lul" to "-ekey" and "-ka"), and sentential endings (from "-nun-" to "-ki-n-"). On the other hand, the order of words within an English sentence is altered in most cases when the sentential form is changed as shown in the sentences (6) and (8).

Given the heavy dependence on syntactic morphemes and less reliance on word order in the Korean syntax, the following specific questions were asked in this study: First, we wondered if Korean syntactic processing also depends on a fronto-temporal network as in other languages. Although several lesion studies on Korean aphasia patients have been conducted and suggested that Korean language also depends on the left temporo-frontal area like other languages [7], there has not been any study particularly focused on the Korean syntactic comprehensive process. A previous study on Japanese-English bilinguals showed that differences in grammatical construction between the two languages affected cortical representation of sentence comprehension. Likewise, we can hypothesize that the cortical representation of Korean syntactic processing may be different from those of other languages [8]. To verify this, we developed a novel test we call the Korean Syntactic Comprehension Test (KSCT). The general strategy of sentence selection and test development was based on the syntactic comprehension battery of Caplan [9], but the syntactic features unique to Korean, such as variations in the postpositional words or sentential endings, scrambling word order within sentences, were strongly emphasized in each item. Then we performed a voxel-based lesion-symptom mapping (VLSM) analysis to know which area of brain is functionally associated with Korean syntactic comprehension ability.

As second, we asked if the KSCT will be useful as other standardized test batteries of Korean language in evaluating the patients with agrammatic aphasia. For this purpose, we analyzed the relationship between patients' performances in the KSCT and in the Korean version-the Western Aphasia Battery (K-WAB), which had been previously validated as a good tool for measuring Korean linguistic capacity [10]. We used two subtests of spontaneous speech and auditory comprehension out of the K-WAB since they were considered to be more related with syntactic comprehension ability than others. When the Western Aphasia Battery was being translated from the original English version to the Korean version, they modified the sentences in the auditory comprehension subtest according to the Korean syntactic rules [11]. As a result, we can consider the auditory comprehension subtest of the K-WAB to reflect the Korean syntactic comprehension ability. In the same way, as the spontaneous speech subtest of the K-WAB also evaluates the Korean grammatical competence [11], we can expect the test result will be correlated with the Korean syntactic comprehension ability. We did not use the subtests evaluating written language areas, however, for the reason that the reading or writing ability could be impaired in stroke patients in addition to the ability of syntactic comprehension.

## 2. Materials and methods

### *2.1. Participants*

A total of 57 subjects participated in the study: 39 patients with ischemic stroke and 18 healthy people as a control group. The patients were recruited and tested at the Department of Neurology in Seoul National University Hospital, Seoul, Republic of Korea. MRI scans and medical records of all the patients were evaluated by a neurologist, and only the patients with acute ischemic stroke within past 2 weeks were included. We excluded the patients with only the lesions attributed to small vessel occlusion [12] or located below tentorium cerebelli. Exclusionary criteria also included confirmed or suspected hearing difficulty, dementia, poor general condition prohibiting test cooperation, and unavailability of MRI scan. Within the patient group, 4 patients had lesions in both hemispheres, 22 patients in left hemisphere only, and 13 patients in right hemisphere only. On clinical assessment, 24 out of 39 patients were considered to have any type of aphasia.

The control group was enrolled from the participating patients' families when possible (most of them were the patients' spouses). As handedness was assessed by the Edinburgh inventory at the outset, only right-handed persons were selected to participate. All subjects were native Korean speakers. Each subject gave written informed consent to the study, which was approved by the Institutional Review Board of Seoul National University Hospital.

### *2.2. Assessment of Korean syntactic comprehension*

The Korean Syntactic Comprehension Test (KSCT) was composed of 42 sentences, each followed by a question that was asking about meaning of the sentence that had just been presented. The question was always expressed in a different grammatical form from the original sentence. Subjects were asked to answer the question "yes" or "no" within a few seconds. Sentential forms of the presented sentences or questions were including affirmative-negative forms, active-passive forms, and simple-compound or complex forms. Common personal names in Korea were used as subjects and objects within the sentences to minimize and normalize semantic processing loads of the sentences. Each question contained different syntactic morphemes from those in the original sentence, and the word order was either kept constant or altered. This ensured that agrammatic comprehenders could make more errors as they tend to interpret a sentence or a question by its canonical word sequence [13].

For example, as shown below, while Question 1 has the similar word sequence to that in Sentence 1, its asking content is the opposite by virtue of the postpositional words ("-ga", "-lul", "-nun"). Agrammatic comprehenders of Korean could incorrectly answer "Yes" to Question 1 as they tend to regard the former (Cheol-su) as a subject and the latter (Yeong-hui) as an object within Question 1.

Sentence 1:
Cheol-su-**ga** Yeong-hui-**lul** do-wa-jun-da.

*subject* *object*

'*Cheol-su* helps *Yeong-hui*.'

Question 1:
Cheol-su-**lul** Yeong-hui-**nun** do-wa-ju-na?

*object* *subject*

'Does *Yeong-hui* help *Cheol-su*?'

Another example is shown with Question 2. While the sequence of the subject and the object is altered (Yeong-hui, Cheol-su to Cheol-su, Yeong-hui), Question 2 is asking the similar content to that in Sentence 2. However, Korean agrammatics could answer "No" to Question 2 for the same reason mentioned above.

Sentence 2:
Yeong-hui-**lul** Cheol-su-**nun** gi-da-rin-da.

*object* *subject*

'*Cheol-su* waits for *Yeong-hui*.'

Question 2:
Cheol-su-**ga** Yeong-hui-**lul** gi-da-ri-na?

*subject* *object*

'Does *Cheol-su* wait for *Yeong-hui*?'

After those sentences and questions were made, they were recorded as a sound file by a professional radio announcer at normal speed. He had not been informed of the experimental conditions. The recordings were then sequenced so that the sentence types were randomized. The subjects were seated in a quiet room, and the nature of the task was explained by a researcher. Two warm-up trials were followed by 42 real trials. Feedback was provided during the warm-up trials to ensure that participants understood the task. Each trial progressed according to the following manner: Through headphones, each subject heard a sentence and, after a 5-second wait, a question. The subject then had 10 seconds to answer. Patients who were not able to pronounce even a monosyllabic word were asked to communicate to the researcher using body language or their own voice signals. When testing patients, the researcher sat behind the subjects so as to avoid inadvertently providing any cues about the sentences. Encouraging feedback was provided periodically to patients, but never corresponded to success or failure on any particular trial. The control subjects were generally given minimal feedback after the warm-up trials and only monitored for general attention and alertness.

The KSCT scores were compared among the patient with clinical aphasia, the patients without clinical aphasia, and the control groups. Within the patient group, 28 subjects' test results of the K-WAB were also available, and 15 out of them were considered to have any degree of agrammatic aphasia. The 28 patients' results of spontaneous speech and auditory comprehension subtest were used in analyzing the correlation with the results of KSCT. Statistical analysis was performed using the ANOVA on ranks and the Pearson's correlation coefficient in SPSS version 11.5. The sensitivity and the specificity of the KSCT in diagnosing agrammatic aphasia were also measured in the 28 patients using the Receiver Operating Characteristic (ROC) curves. Using the cutoff KSCT score calculated from the ROC analysis, the aphasic patients were also divided into two groups with normal and abnormal KSCT results.

### 2.3. *Voxel-based lesion-symptom mapping* (*VLSM*)

Brain MR images of the patients were analyzed using VLSM in the search for lesion correlations with the test results. As the time gap between the KSCT and MRI was no more than 2 weeks for any patient, only acute lesion was considered to be significant. To define location and size of the acute ischemic lesions, diffusion weighted images taken within 2 weeks from the onset of symptom were used. Lesions were identified from the diffusion weighted images, and drawn manually slice by slice on the ch2 template using free MRIcron software (by Chris Rorden, *http://www.sph.sc.edu/comd/rorden/mricron/*) by a board-certified neurologist, and then saved as volumes of interest. Initially, each patient's drawn lesion image was simply overlaid onto the ch2 template, and then entered the VLSM analysis together with the KSCT score.

The basic statistical process carried out in VLSM is summarized as follows: at each voxel, patients were divided into two groups according to whether they did or did not have a lesion affecting that voxel. Behavioral scores were then compared between these two groups using analysis of covariance (ANCOVA) with age as covariate (as only age showed significant correlation with KSCT score), and the resultant $p$-values were overlaid onto the ch2 template as a color map. VLSM algorithms were programmed in MATLAB (The Mathworks, Inc.) and are available online at *http://crl.ucsd,edu/vlsm*. Multiple comparisons were controlled using the expected proportion of false positives (FDR correction) [14,15].

Involvement of each voxel was also compared between aphasic and non-aphasic patients using Liebermeister measure of NPM, which is another software of VLSM (by Chris Rorden, http://www.sph.sc.edu/comd/rorden/mricron/stats.html). Within the aphasic patients, the patients with KSCT scores below the cutoff value were also compared with the other patients with higher KSCT scores using the similar method.

## 3. Results

The KSCT score and the demographic characteristics of each group are shown in Table 2. Within the patient group, the aphasic patients showed significantly lower performance than the control group in the KSCT while the non-aphasic patients showed almost similar level of performance to the control group. No significant difference was noticed among the groups with respect to age, sex, and education. Within the patient group, KSCT score showed negative correlation with age ($r = -0.418$, $p = 0.008$) whereas it did not show

Table 2
Demographic characteristics and KSCT score of each group

| | Stroke patients ($n = 39$) | | Normal controls ($n = 18$) | $p$ value |
|---|---|---|---|---|
| | Aphasic ($n = 24$) | Non-aphasic ($n = 15$) | | |
| Age (years) | 62.7 (11.2) | 59.3 (14.1) | 57.8 (11.4) | 0.404 |
| Sex (% Male) | 75.0 | 53.3 | 63.6 | 0.062 |
| Education (years) | 11.8 (4.6) | 9.3 (6.0) | 10.8 (4.9) | 0.417 |
| KSCT score | 17.8 (12.6)* | 27.3 (7.4) | 29.6 (5.2) | 0.002 |

All continuous variables are expressed as mean (SD).
KSCT: Korean Syntactic Comprehension Test.
*Statistically significant when compared with the normal control group.

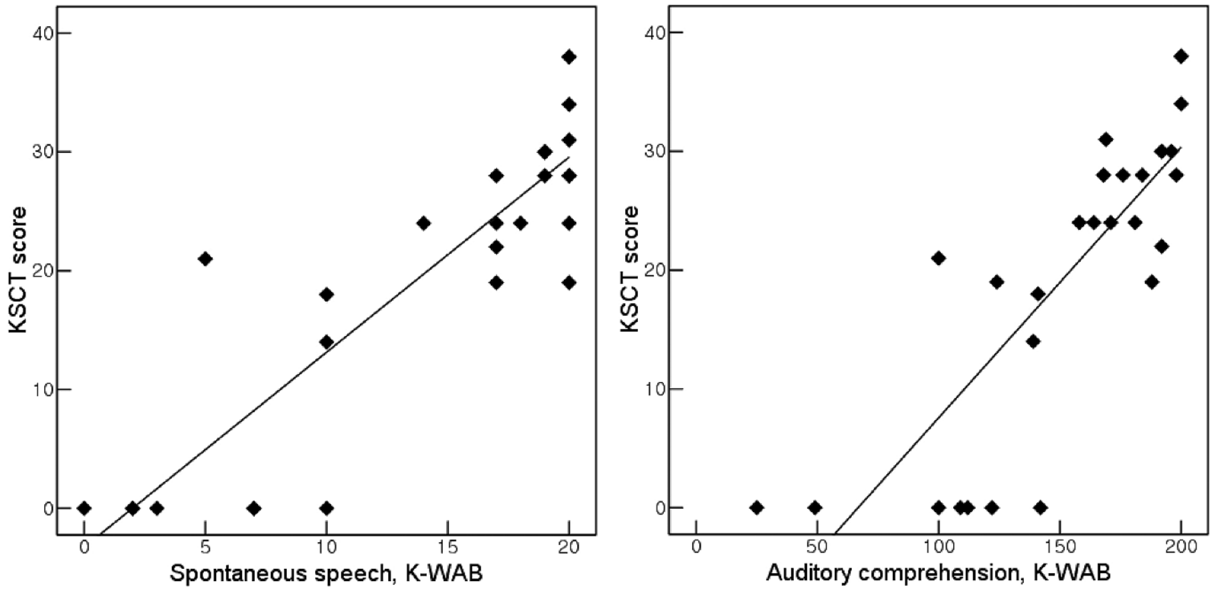

Fig. 1. Correlations between the results of the KSCT and each subtest of the K-WAB: Spontaneous speech (left) and Auditory comprehension (right).

any significant association with sex or education period.

When the correlation coefficients were calculated between the KSCT scores and each of the two subtest scores in K-WAB, close correlations were observed in both cases (with the spontaneous speech subtest: $r = 0.880$, $p < 0.001$, with the auditory comprehension subtest: $r = 0.826$, $p < 0.001$, see Fig. 1). With the ROC analysis, the area under the curve was 0.75, which means that the KSCT has significant diagnostic value in differentiating agrammatic aphasics from other patients with ischemic stroke. With the cutoff value of 7.5, its sensitivity and specificity was 100.0% and 75.0%, respectively.

Figure 2 shows an overlay map for the lesions of the patients in this study, depicting areas where the majority of the patients were lesioned. The most commonly involved area was the bilateral basal ganglia and corona radiata, which means that statistical power was greatest in that area. On the other hand, the anterior frontal or occipital regions were rarely involved in the participating patients.

The VLSM results of the patients group are shown in Fig. 3. As the effect of age was factored out using ANCOVA, the color map of $p$ value demonstrated that lower KSCT score was associated mainly with the lesions involving left perisylvian and anterior temporal areas. These areas included the left superior and middle frontal gyrus, frontal operculum, insular cortex and adjacent subcortical areas, anterior portion of superior temporal gyrus, middle and inferior temporal gyrus. Although a few right hemispheric lesions were also associated with lower KSCT score, their statistical significance was relatively low.

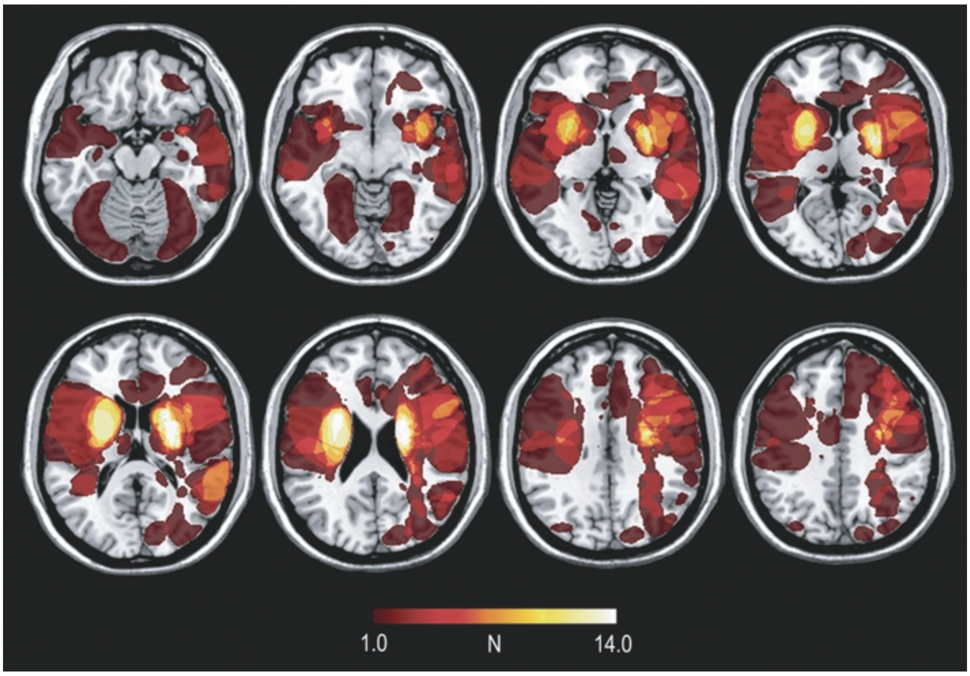

Fig. 2. Overlay plots of all acute ischemic lesions in the patients. Higher values represent voxels where a larger number of patients were lesioned.

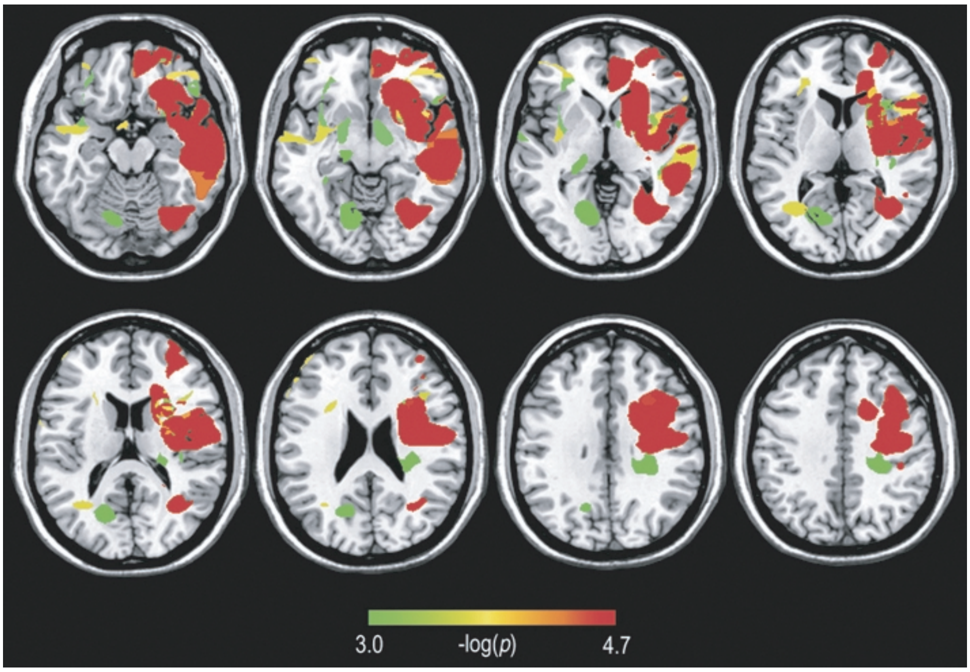

Fig. 3. Representative slices from maps of voxel-by-voxel ANCOVAs covarying out age. One-tailed $p$-values are plotted. The values displayed at each voxel are – log($p$) values comparing the patients lesioned at that voxel to the patients intact at that voxel. Low $p$ values top the scale in red, indicating areas where damage led to more significant deficits in KSCT performance.

When the 24 aphasic and 15 non-aphasic patients were compared at each voxel using VLSM, there was no statistically significant area where the aphasic patients were more likely to be lesioned than the non-aphasic patients. Within the 24 aphasic patients, however, 7 patients with KSCT scores below the cutoff value showed more involvement of the left basal ganglia and the left corona radiata when compared to the other 17 patients with higher KSCT scores.

## 4. Discussion

On the basis of those results, we conclude that Korean syntactic processing depends on a fronto-temporal network including left perisylvian and anterior temporal areas like other languages. A vast number of previous lesion studies have indicated that the left anterior brain regions are involved in syntactic processing, and the left temporal regions support lexical-semantics [1, 16,17]. More recent studies have posited that the syntactic rule system is supported by a procedural memory circuit located in the frontal cortex and basal ganglia whereas the lexicon is represented in a temporo-parietal circuit [18,19]. It has also been demonstrated by an event-related functional magnetic imaging (fMRI) study that the processing of syntactic violations in a sentence mainly relies on the anterior portion of the superior temporal gyrus, the left posterior frontal operculum, and the left basal ganglia, while semantic violations involve the middle and posterior portion of the bilateral superior temporal region and the insular cortex [4]. These indicate that both semantic and syntactic processes are supported by a fronto-temporal network with distinct areas specialized for each of the processes. Our results are in line with these previous findings, despite the distinct features of Korean syntax as described in Introduction.

In addition, our data suggested that KSCT performance is also associated with the left medial frontal, inferior parietal area, and some part of the right perisylvian area. According to previous studies, the cingulate gyrus and nearby regions of medial frontal lobe have showed increased blood flow when subjects processed syntactically [20,21]. Activation has also been found in parietal lobe on syntactic task in several studies [22]. These activations have been attributed to non-domain-specific attentional processes (cingulate and midline frontal activation), and to visual-spatial processing (parietal activation), but they may reflect syntactic processing [13]. There also have been some reports supporting a role for non-dominant hemisphere homologues of Broca's and Wernicke's areas in syntactic comprehension: these areas increased their BOLD signal in response to processing syntactically complex sentences, to a lesser extent than dominant hemisphere [23]. It has also been suggested that subcortical structures are involved in rule-based processing in language, which is consistent with our results in the left basal ganglia and corona radiata in the aphasic patients with lower KSCT scores [19,24]. However, the possibility also should be considered that the relatively high statistical power in the basal ganglia, as shown in Fig. 2, could result in false-positive findings.

In the present study, KSCT score could clearly discriminate the aphasic patients with ischemic stroke from the normal subjects. Moreover, KSCT score showed high correlation with both the spontaneous speech and auditory comprehension subtests scores in the K-WAB, which is supposed to be the gold standard of language evaluation methods in Korea. Using the K-WAB as a standard, in addition, the sensitivity and the specificity of the KSCT in discriminating agrammatic aphasia from other stroke patients were also high enough to support the diagnostic value.

Given the limitations of the current study in terms of its small sample size and limited availability of K-WAB results, further research is needed to validate the current findings and assess the clinical value of the KSCT. It will be also worthwhile to modify the items of KSCT being based on the linguistic hypotheses about Korean syntactic comprehension that have been recently suggested [5,6]. Another limitation of the KSCT is that it is an auditory-only test so that impairment in any step of auditory or comprehensive language processes could influence the result. Thus, it should be always used in combination with other testing methods including the K-WAB, although only a few subtests of K-WAB were available in this study from the poor condition of acute stroke patients. Taking into account the unique features of Korean, however, this study is still significant since there has not been any valuable tool suitable for evaluating comprehension abilities of Korean syntactic structures.

In conclusion, Korean syntactic comprehension depends mainly on left perisylvian and anterior temporal region, which is the same finding with other languages in which word order determines syntax – although Korean syntactic parsing is primarily accomplished by morphemes added at the end of content words. Also, the KSCT is valuable for assessing left fronto-temporal functioning and syntactic comprehension capability of Korean patients, despite the unique syntactic features of the language.

## Acknowledgements

This research was supported by a grant from National Research Foundation of Korea (No.20090080348). We would also like to thank Jeong-Tae Kim, Woong Jang, and other staffs of Korean Broadcasting System (KBS) for technical assistance.
